# Identification of Candidate Casein Kinase 2 Substrates in Mitosis by Quantitative Phosphoproteomics

**DOI:** 10.3389/fcell.2017.00097

**Published:** 2017-11-22

**Authors:** Scott F. Rusin, Mark E. Adamo, Arminja N. Kettenbach

**Affiliations:** ^1^Department of Biochemistry and Cell Biology, Geisel School of Medicine at Dartmouth, Hanover, NH, United States; ^2^Norris Cotton Cancer Center, Dartmouth-Hitchcock Medical Center, Lebanon, NH, United States

**Keywords:** CK2, proteomics, CX-4945, mitosis, phosphoproteomics, chromosome condensation

## Abstract

Protein phosphorylation is a crucial regulatory mechanism that controls many aspects of cellular signaling. Casein kinase 2 (CK2), a constitutively expressed and active kinase, plays key roles in an array of cellular events including transcription and translation, ribosome biogenesis, cell cycle progression, and apoptosis. CK2 is implicated in cancerous transformation and is a therapeutic target in anti-cancer therapy. The specific and selective CK2 ATP competitive inhibitor, CX-4945 (silmitaseratib), is currently in phase 2 clinical trials. While many substrates and interactors of CK2 have been identified, less is known about CK2 substrates in mitosis. In the present work, we utilize CX-4945 and quantitative phosphoproteomics to inhibit CK2 activity in mitotically arrested HeLa cells and determine candidate CK2 substrates. We identify 330 phosphorylation sites on 202 proteins as significantly decreased in abundance upon inhibition of CK2 activity. Motif analysis of decreased sites reveals a linear kinase motif with aspartic and glutamic amino acids downstream of the phosphorylated residues, which is consistent with known substrate preferences for CK2. To validate specific candidate CK2 substrates, we perform *in vitro* kinase assays using purified components. Furthermore, we identified CK2 interacting proteins by affinity purification-mass spectrometry (AP-MS). To investigate the biological processes regulated by CK2 in mitosis, we perform network analysis and identify an enrichment of proteins involved in chromosome condensation, chromatin organization, and RNA processing. We demonstrate that overexpression of CK2 in HeLa cells affects proper chromosome condensation. Previously, we found that phosphoprotein phosphatase 6 (PP6), but not phosphoprotein phosphatase 2A (PP2A), opposes CK2 phosphorylation of the condensin I complex, which is essential for chromosome condensation. Here, we extend this observation and demonstrate that PP6 opposition of CK2 is a more general cellular regulatory mechanism.

## Introduction

Cells traversing mitosis undergo extraordinary changes in organization in order to equally divide genetic material and organelles between two daughter cells. In part, these changes are regulated by reversible phosphorylation by kinases and phosphatases. Over the course of their lifetime, at least three-quarters of all proteins are phosphorylated on one or more serine, threonine, or tyrosine residues (Cohen, 2000; Sharma et al., 2014). A tug-of-war between the opposing activities of kinases and phosphatases ensures the proper timing and coordination of events during mitotic progression. Control of nuclear envelope breakdown, golgi vesiculation and reformation, and chromosome condensation and segregation are key to a successful mitosis, and misregulation of any of these events can be detrimental to cells (Ottaviano and Gerace, 1985; Abe et al., 2011; Zhang et al., 2014). For instance, chromosome missegregation can lead to aneuploidy, a hallmark of human cancer (Rajagopalan and Lengauer, 2004; Hanahan and Weinberg, 2011). Although the majority of protein phosphorylation in mitosis has been attributed to master mitotic regulators, such as CDK1, Plk1, and Aurora A/B (Salaun et al., 2008), more recently roles for Casein kinase 2 (CK2) in the regulation of cell cycle transitions and mitosis have emerged (Takemoto et al., 2006; Yde et al., 2008; St-Denis et al., 2009, 2011; Li et al., 2010; Barrett et al., 2011; Peng et al., 2011).

Protein kinase CK2 is a highly conserved and constitutively expressed and active serine/threonine kinase found in all eukaryotes (Litchfield, 2003). CK2 regulates many cellular functions, including gene expression, translation, cell cycle progression and survival (Litchfield and Lüscher, 1993; Pinna and Meggio, 1997; Ahmed, 1999; Guerra and Issinger, 1999; Ahmed et al., 2002). CK2 is also overexpressed in a number of malignancies, and is a promising therapeutic target for several forms of cancer (Tawfic et al., 2001; Perea et al., 2008; Hanif et al., 2010; Siddiqui-Jain et al., 2010). In humans, CK2 is a heterotetrameric enzyme consisting of two catalytic α subunits (αα, αα,′ or α′α′) and two regulatory β subunits (Niefind et al., 2001). While many substrates of CK2 have been described, less is known about CK2 substrates in mitosis specifically.

Casein kinase 2 (CK2) has been implicated as a regulator of chromosome condensation (Takemoto et al., 2006). CK2 was shown to phosphorylate condensin I, a pentameric protein complex involved in chromosome compaction during mitosis (Takemoto et al., 2006). This phosphorylation inhibits condensin I activity in interphase until the G2/M transition, when these phosphorylation sites are removed by the phosphoprotein phosphatase PP6, resulting in condensin I activation (Rusin et al., 2015). Furthermore, CK2 has been shown to be necessary for the G1/S and G2/M transitions in *Saccharomyces cerevisiae*, and depletion of CK2 blocks cell cycle progression (Pepperkok et al., 1991, 1994; Lorenz et al., 1993; Glover, 1998). In mitosis, CK2 has been shown to phosphorylate Cdc25B, phosphoprotein phosphatase 2A (PP2A), HDAC1/2, and Topoisomerase IIα/β (Hériché et al., 1997; Daum and Gorbsky, 1998; Escargueil et al., 2000; Theis-Febvre et al., 2003; Khan et al., 2013).

In recent years, many substrates of mitotic kinases have been identified through the use of specific and selective inhibitors combined with mass spectrometry-based phosphoproteomics (Kettenbach et al., 2011; Peng et al., 2011; Santamaria et al., 2011; Petrone et al., 2016). In this study we used a combination of the selective CK2 inhibitor CX-4945 and mass spectrometry-based phosphoproteomics as well as affinity purification—mass spectrometry (AP-MS) to systematically identify candidate CK2 substrates and CK2-dependent biological processes in mitotically-arrested HeLa cells. We validate specific candidate CK2 substrates *in vitro*, elaborate the role of CK2 in chromosome condensation in cells, and demonstrate a more general mechanism for phosphoprotein phosphatase 6 (PP6) opposition of CK2 phosphorylation.

## Materials and methods

### Cells

HeLa and HEK-293T cells were grown as adherent cultures in Dulbecco's modified Eagle's media (DMEM, Cellgro Mediatech Inc.,) with 8% heat-inactivated FetalPlex (Gemini) and penicillin-streptomycin (100 U/ml and 100 μg/ml, respectively; Cellgro Mediatech Inc) at 37°C in a humidified incubator with 5% CO_2_. Sf9 cells were grown in Grace's supplemented insect cell media (LifeTechnologies) with 10% heat-inactivated fetal bovine serum (FBS) (Hyclone), 10 μg/ml gentamycin (SIGMA), and 0.25 μg/ml amphotericin B (SIGMA). Sf9 cells were maintained at 28°C in a non-humidified incubator.

For SILAC analysis, HeLa cells were grown in heavy or light DMEM (GIBCO) supplemented with 10% dialyzed FBS (Hyclone) and penicillin-streptomycin. “Heavy” media contained 100 mg/L ^13^C_6_^15^N_2_-lysine and 100 mg/L ^13^C_6_^15^N_4_-arginine (Cambridge Isotope Laboratories), while “light” media contained 100 mg/L ^12^C_6_^14^N_2_-lysine and 100 mg/L ^12^C_6_^14^N_4_-arginine (Sigma). Cells were grown for a minimum of six doublings in the respective medium.

To synchronize cells in mitosis, thymidine (1 mM, Sigma) was added for 22 h to both conditions, followed by a 3 h washout with PBS (Corning) and subsequent addition of Taxol (Sigma) for 16 h.

### Phosphoproteome-wide analysis of CK2 inhibition

For phosphoproteomic analysis, heavy and light cells were arrested in mitosis and treated with MG132 at a concentration of 10 μM for 30 min. Heavy-labeled HeLa cells were then treated with 5 μM CX-4945, while light-labeled HeLa cells were treated with DMSO and incubated at 37°C for 45 min. Mitotic HeLa cells were collected by mitotic shake-off, washed with PBS, snap frozen in liquid nitrogen, and stored at −80°C. Experiments were performed in biological triplicate. HeLa cell pellets were thawed on ice and lysed in ice-cold lysis buffer [8 M urea, 25 mM Tris-HCl pH 8.6, 150 mM NaCl, phosphatase inhibitors (2.5 mM beta-glycerophosphate, 1 mM sodium fluoride, 1 mM sodium orthovanadate, 1 mM sodium molybdate) and protease inhibitors (1 mini-Complete EDTA-free tablet per 10 ml lysis buffer; Roche Life Sciences)] and sonicated three times for 15 s each with intermittent cooling on ice. Lysates were centrifuged at 15,000 × *g* for 30 min at 4°C. Supernatants were transferred to a new tube and the protein concentration was determined using a BCA assay (Pierce/ThermoFisher Scientific). For reduction, DTT was added to the lysates to a final concentration of 5 mM and incubated for 30 min at 55°C. Afterwards, lysates were cooled to room temperate and alkylated with 15 mM iodoacetamide at room temperature for 45 min. The alkylation was then quenched by the addition of an additional 5 mM DTT. After 6-fold dilution with 25 mM Tris-HCl pH 8, the samples were digested overnight at 37°C with 2.5% (w/w) trypsin. The next day, the digest was stopped by the addition of 0.25% TFA (final v/v), centrifuged at 3,500 × *g* for 30 min at room temperature to pellet precipitated lipids, and peptides were desalted on a 500 mg (sorbent weight) SPE C_18_ cartridge (Grace-Davidson). Peptides were lyophilized and stored at −80°C until further use.

### Phosphopeptide enrichment

Phosphopeptide purification was performed as previously described (Kettenbach and Gerber, 2011). Briefly, peptides were resuspended in 2 M lactic acid in 50% ACN (“binding solution”). Titanium dioxide microspheres were added and vortexed by affixing to the top of a vortex mixer on the highest speed setting at room temperature for 1 h. Afterwards, microspheres were washed twice with binding solution and three times with 50% ACN/0.1% TFA. Peptides were eluted twice with 50 mM KH_2_PO_4_ (adjusted to pH 10 with ammonium hydroxide). Peptide elutions were combined, quenched with 50% ACN/5% formic acid, dried and desalted on a μHLB OASIS C_18_ desalting plate (Waters).

### Pentafluorophenyl-based reversed phase HPLC

Offline PFP-based reversed phase HPLC fractionation was performed as previously described (Grassetti et al., 2017). Briefly, phosphopeptides were fractionated using a Waters XSelect HSS PFP 2.5 μm 2.1 × 150 mm column on an Agilent 1100 liquid chromatography system, buffer A was 3% acetonitrile/0.1% TFA, and buffer B was 95% acetonitrile/0.1% TFA. Flow rate was 150 μl/min with a constant column temperature of 20°C. Phosphopeptides were fractioned using a 60 min linear gradient from 8 to 45% acetonitrile and collected as 48 fractions between minutes 2 and 65, the 48 fractions were then combined into 24 total samples prior to drying in a SpeedVac and LC-MS/MS analysis.

### LC-MS/MS analysis

LC-MS/MS analysis for peptides and phosphopeptides was performed on a Q-Exactive Plus hybrid quadrupole Orbitrap mass spectrometer (Thermo Fisher Scientific, Bremen, Germany) equipped with an Easy-nLC 1000 (Thermo Fisher Scientific) and nanospray source (Thermo Fisher Scientific). Phosphopeptides were redissolved in 5% ACN/1% formic acid and loaded onto a trap column at 2,500 nl/min (1.5 cm length, 100 μm inner diameter, ReproSil, C_18_ AQ 5 μm 200 Å pore; Dr. Maisch, Ammerbuch, Germany) vented to waste via a micro-tee and eluted across a fritless analytical resolving column (35 cm length, 100 μm inner diameter, ReproSil, C_18_ AQ 3 μm 200 Å pore) pulled in-house (Sutter P-2000, Sutter Instruments, San Francisco, CA) with a 60 min gradient of 5–30% LC-MS buffer B (LC-MS buffer A: 0.0625% formic acid, 3% ACN; LC-MS buffer B: 0.0625% formic acid, 95% ACN). The Q-Exactive Plus was set to perform an Orbitrap MS1 scan (*R* = 120 K; AGC target = 2.5e5) from 350 to 1,500 Thomson, followed by HCD MS2 spectra on the most abundant precursor ions detected by Orbitrap scanning (*R* = 15 K; AGC target = 40,000; max ion time = 50 ms) for 2.5 s before repeating the cycle. Precursor ions were isolated for HCD by quadrupole isolation at width = 0.6 Thomson, and HCD fragmentation at 30% collision energy. Charge state 2, 3, and 4 ions were selected for MS2.

### Peptide spectral matching and bioinformatics

Raw data were searched using COMET (release version 2014.01) in high resolution mode (Eng et al., 2013) against a target-decoy (reversed) (Elias and Gygi, 2007) version of the human proteome sequence database (UniProt; downloaded 2/2013, 40,482 entries of forward and reverse protein sequences) with a precursor mass tolerance of ±1 Da and a fragment ion mass tolerance of 0.02 Da, and requiring fully tryptic peptides (K,R; not preceding P) with up to three miscleavages. Static modifications included carbamidomethylcysteine and variable modifications included: oxidized methionine, heavy lysine and arginine, phosphorylated serine, threonine, and tyrosine. Searches were filtered using orthogonal measures including mass measurement accuracy (±3 ppm), Xcorr for charges from +2 through +4, and dCn targeting a <1% FDR at the peptide level. The probability of phosphorylation site localization was assessed using PhosphoRS (Taus et al., 2011). Quantification of LC-MS/MS spectra was performed using MassChroQ (Valot et al., 2011). Phosphopeptide ratios were adjusted for mixing errors based on the median of the log_2_ H/L distribution.

### Statistical rationale and data, motif analysis

Phosphopeptides were filtered by their H/L log_2_ ratio averages <-1 and the corresponding *p* < 0.05, which were calculated using a two tailed Student's *t*-test assuming unequal variance. These peptides were then subjected to motif determination using the Motif-X algorithm and visualized using Weblogo 3.0 (Crooks et al., 2004; Schwartz and Gygi, 2005; Chou and Schwartz, 2011). Motifs were generated using a 10% threshold for input data. Protein-protein interactions of proteins belonging to phosphopeptides with significant increase in phosphorylation occupancy were determined using the STRING database and analyzed in Cytoscape (Shannon et al., 2003; Szklarczyk et al., 2015). Edges represent protein-protein interactions based on the STRING database. GO analyses were performed in Cytoscape using BiNGO to test for ontology enrichment of biological processes and cellular components (Saito et al., 2012). To assess significance of enrichment of terms, a hypergeometric test and Benjamini & Hochberg false discovery rate (FDR) correction were used. For a processes or component to be considered as “enriched,” a corrected *P*-value cutoff of 0.05 was applied.

### CK2α/β cloning, expression and insect cell purification

CK2α and CK2β were amplified via PCR using the following primers:

**Table:** 

CK2α F	5′-ACGCGTCGACATGTCGGGACCCGTGCCAAG-3′
CK2α R	5′-CCCAAGCTTTTACTGCTGAGCGCCAGCG-3′
CK2β F	5′-CGCGGATCCATGAGCAGCTCAGAGGAGGTG-3′
CK2β R	5′-CCGCTCGAGTCAGCGAATCGTCTTGACTG-3′

Both genes were cloned into the pFastBac1 (Life Technologies) vector by restriction digest. CK2α was cloned into a pFastBac1 vector containing a 10-histidine tag while CK2β was cloned into pFastBac1 with no tag. Constructs were sequenced and transformed into the DH10α *E. coli* strain to create bacmids. The resulting bacmid DNA was isolated, genotyped, and transfected into Sf9 cells to create recombinant baculovirus. For protein production, Sf9 cells were coinfected in a T75 dish with CK2α-10his and CK2β viruses for 84 h. Prior to collection, cells were treated with okadaic acid for 2 h, collected, and washed in PBS. Cells were lysed in lysis buffer containing: 20 mM HEPES pH 7.5, 150 mM NaCl, 5 mM β-glycerphosphate, 2 mM sodium fluoride, 2 mM sodium molybdate, 1 mM sodium orthovanadate, 1% CHAPS, 2.5 mM EGTA, 5 mM BME, 25 mM imidazole and a protease inhibitor tablet. Samples were sonicated three times for 15 s each. Cell lysate was clarified at 8,000 × *g* at 4°C for 30 min. Nickel-NTA agarose resin previously washed in lysis buffer was added to the clarified lysate for 2 h with rotation at 4°C. Kinase complex bound nickel-NTA agarose was washed twice with lysis buffer, twice with wash buffer (lysis buffer with additional 500 mM NaCl), followed by elution in 200 μL of elution buffer (lysis buffer with additional 400 mM imidazole and 1 mM EDTA). Elutions were dialyzed against *in vitro* kinase assay buffer containing 5 mM HEPES pH 7.5, 125 mM NaCl, 0.25 mM EDTA, and 0.5 mM DTT.

### CK2 subunit cloning for immunoprecipitation

Subunits of CK2 were amplified via PCR using the following primers:

**Table:** 

CK2α	5′-CCCAAGCTTATGTCGGGACCCGTGCCAAGCAGG-3′
CK2α	5′-CGCGGATCCTTACTGCTGAGCGCCAGCGGCAG-3′
CK2β	5′-CCCAAGCTTATGAGCAGCTCAGAGGAGGTGTC-3′
CK2β	5′-CGCGGATCCTCAGCGAATCGTCTTGACTGGGCTC-3′
CK2α′	5′-CCCAAGCTTATGCCCGGCCCGGCCGCGG-3′
CK2α′	5′-CGCGGATCCTCATCGTGCTGCCGTGAGACCAC-3′

All genes were cloned into the p3xFLAG-CMV-10 (SIGMA) vector using restriction digests. Constructs were sequenced to confirm identities. Vectors were then transfected into HEK-293T cells which were subsequently arrested in mitosis as described above. Cells were lysed in FLAG lysis buffer containing 50 mM Tris-HCl pH 7.5, 150 mM NaCl, 1% Triton-X100 and a protease inhibitor tablet. Samples were sonicated three times for 15 s each. Cell lysate was clarified at 8,000 × *g* at 4°C for 15 min. Anti-FLAG M2 affinity gel (SIGMA) previously washed in FLAG lysis buffer was applied to the clarified lysate for 2 h with rotation at 4°C. Bound resin was then washed three times with FLAG lysis buffer and eluted in 30 μl TBS with 0.16 μg/μl 3xFLAG peptide by vortexing gently for 30 min at 4°C. Elutions were then Trichloroaceitic acid (TCA) precipitated. Briefly, samples were diluted in 20% TCA, incubated on ice for 15 min, and centrifuged at 21,100 × *g* for 15 min at 4°C. Samples were resuspended in 10% TCA and centrifuged at 21,100 × *g* for 10 min at 4°C. This step was repeated once more. Samples were then resuspended in cold acetone and centrifuged at 21,100 × *g* for 10 min at 4°C. This step was repeated once more. Samples were air-dried and processed for LC-MS/MS analysis.

### Purification of substrates from *E. coli*

Substrates were amplified via PCR using the following primers:

**Table:** 

EF1D F	5′-CCGCTCGAGGCTACAAACTTCCTAGCAC-3′
EF1D R	5′-AAGGAAAAAAGCGGCCGCTCAGATCTTGTTGAAAG-3′
HAP28 F	5′-CCGCTCGAGCCTAAAGGAGGAAGAAAG-3′
HAP28 R	5′-AAGGAAAAAAGCGGCCGCTTACTTATTCAGGGAG-3′
S30BP F	5′-CCGCTCGAGGCGGGGAAGAAGAATGTTC-3′
S30BP R	5′-AAGGAAAAAAGCGGCCGCTCACTGCTTGGCCTTC-3′

Each substrate was cloned into the pET16b vector containing a 10x-His tag. The constructs were sequenced and transformed into BL21 (DE3) pLys *E. coli*. Colonies were grown overnight in LB liquid medium containing 0.1 mg/mL of ampicillin at 37°C to saturation. Cultures were then diluted into LB liquid medium containing 0.1 mg/mL ampicillin and grown at 37°C until an OD600 reading of 0.6. Cultures were then induced with 1 mM IPTG and moved to 18°C overnight. Soluble proteins were purified under native conditions. Pellets were resuspended in ~6–7 mL of lysis buffer containing: 50 mM NaH_2_PO_4_, 300 mM NaCl, and 10 mM imidazole pH 8. Cells were sonicated, pre-cleared, and incubated with nickel-NTA beads (Qiagen) for 3 h at 4°C. Beads were collected and washed in wash buffer containing: 50 mM NaH_2_PO_4_, 300 mM NaCl, 20 mM imidazole pH 8. The beads were eluted with 100 μL of elution buffer containing: 50 mM NaH_2_PO_4_, 300 mM NaCl, 500 mM imidazole pH 8. Purified protein was dialyzed overnight in dialysis buffer containing: 10 mM HEPES-KOH pH 7.7, 100 mM NaCl, 1 mM DTT, 0.1 mM EDTA, 10% glycerol.

### CK2 *in vitro* kinase assay

Kinase assays containing 75 ng of CK2α/β, 1 μg of purified substrate, 50 mM HEPES pH 7.5, 10 mM NaCl, 2 mM DTT, 1 mM MnCl_2_, 0.01% Brij 35 and 100 μM ATP were placed at 28°C for 2 h, followed by addition of 50 mM Tris-HCl, pH 8.6 containing 1% SDS. Reactions were dialyzed overnight against *in-vitro* kinase assay buffer without ATP to inactive CK2 in reactions.

### PP6 *in vitro* phosphatase assay

HEK-293T cells were transfected with p3xFlag-CMV10-PP6c and stable cells lines were selected using G418. Purification of PP6c holoenzymes was performed using anti-Flag M2 affinity gel (SIGMA) (15 μl resin for each 15 cm tissue culture dish lysed) and eluted with 3xFLAG-peptide (final concentration 150 ng/μl). For dephosphorylation reactions, substrates were incubated in the presence or absence of PP6c holoenzymes in phosphatase buffer (50 mM HEPES pH 7.5, 10 mM NaCl, 2 mM DTT, 1 mM MnCl_2_, 0.01% Brij 35) and incubated for 2 h at 28°C. Reactions were quenched by the addition of SDS-PAGE sample buffer, reduced and alkylated (as described above), and separated by SDS-PAGE gel electrophoresis. Substrate bands were excised and digested with trypsin (for EF1D, HAP28, and S30BP) in 25 mM ammonium bicarbonate overnight at 37°C or Proteinase K (for NCAP-D2) in 50 mM TEAB for 1 h at 37°C. Peptides were extracted using 5% formic acid/50% ACN and dried. NCAP-D2 peptides were labeled heavy and light by reductive dimethylation (as described above), mixed, and desalted. Peptides were analyzed on a Q-Exactive Plus mass spectrometer (Thermo Scientific) or Orbitap Fusion mass spectrometer equipped with an Easy-nLC 1000 (Thermo Scientific). Raw data were searched using COMET in high resolution mode (Eng et al., 2013) against a condensin I sequence database with a precursor mass tolerance of ±20 ppm, no enzyme specificity, carbamidomethylcysteine, and dimethylation at peptide amino-termini and lysines, as fixed modifications. Oxidized methionine, phosphorylated serine, threonine and tyrosine, isotopically heavy label (+ 8.04437) at peptide amino-termini and lysines, were searched as variable modifications. Probability of phosphorylation site localization was determined by PhosphoRS (Taus et al., 2011). Quantification of LC-MS/MS spectra was performed using MassChroQ (Valot et al., 2011).

### Chromosome spreads

CK2α expression in HeLa-FLP was induced by addition of doxycycline for 48 h, and then cells were treated for 4 h with 100 ng/mL nocodazole. Cells were collected by mitotic shake-off and pelleted. Cells were resuspended gently in 75 mM KCl, incubated at room temperature for 5 min and at 4°C for 1 min and pelleted by centrifugation. Cells were fixed with methanol:acetic acid (3:1) and pelleted. Cells were washed twice with methanol:acetic acid (3:1), resuspended and dropped onto glass slides. Slides were allowed to dry and DNA was stained with NucBlue Fixed Cell ReadyProbes reagent (Life Technologies) diluted in PBS for 30 min and sealed with coverslips using ProLong Gold (Life Technologies). Experiments were done in biological triplicates. At least 300 chromosome spreads were counted per condition and experiment.

## Results

### Phosphoproteomic profiling of mitotic HeLa cells treated with CX-4945

**Figure 1:**
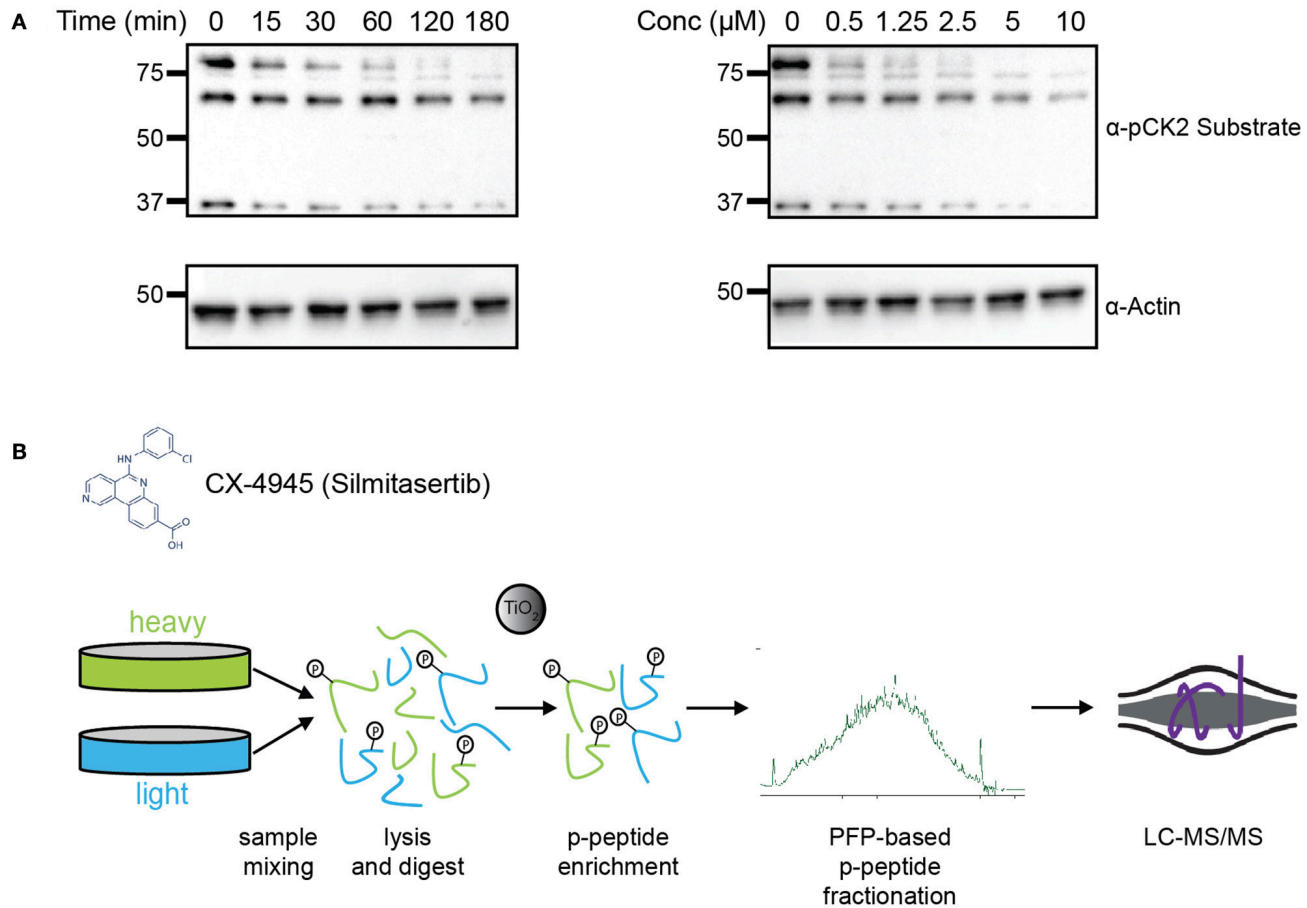
Experimental design for identification of CK2 targets in mitotically arrested HeLa cells. **(A)** Time course and dosage titration of CX-4945 in mitotically arrested HeLa cells. Cells were treated at 5 μM for times shown or for 45 min with concentrations indicated, collected, and analyzed by western blot using α-phospho-CK2 substrate (pS/pT-D-X-E) or α-actin antibodies. **(B)** Phosphoproteomic screen scheme for identification of mitotic substrates. Briefly, cells were treated with vehicle control or CX-4945, mixed, lysed, reduced, alkylated, and digested with trypsin. Peptides were then phospho-enriched using titanium dioxide microspheres, separated using pentafluorophenyl reverse-phase fractionation and analyzed by LC-MS/MS.

To determine the efficacy of CX-4945 in inhibiting CK2, HeLa cells were treated cells with either various concentrations of CX-4945 for 1 h or at a concentration of 5 μM for different lengths of time to establish an optimal dose and time for treatment (Figure 1A). Cells were collected, lysed and analyzed by western blotting using an antibody against the pan-CK2 phosphorylated consensus motif. Based on this analysis, as well as previously published reports, we chose to treat cells with 5 μM CX-4945 for 45 min (Siddiqui-Jain et al., 2010). To identify candidate CK2 substrates, HeLa cells were metabolically-labeled using growth media containing light or heavy arginine and lysine (SILAC) (Figure 1B; Supplementary Figure 1; Ong et al., 2002), synchronized in the cell cycle with a thymidine block, released from the block and arrested in mitosis using the microtubule stabilizer Taxol. Mitotically-arrested cells were treated with the proteasome inhibitor MG-132 for 30 min to block protein degradation upon inhibitor addition. Subsequently, heavy-labeled cells were treated with CX-4945 for 45 min, while light-labeled cells were control-treated with DMSO. After 45 min, cells were collected, mixed 1:1 based on cell counting, lysed and protein digested with trypsin. Phosphopeptides were enriched using titanium dioxide microsphere enrichment, fractionated by pentafluorophenyl reverse-phase separation and analyzed by liquid chromatography coupled to tandem mass spectrometry (LC-MS/MS) (Figure 1B). These phosphoproteomic experiments were conducted in biological triplicates.

**Figure 2:**
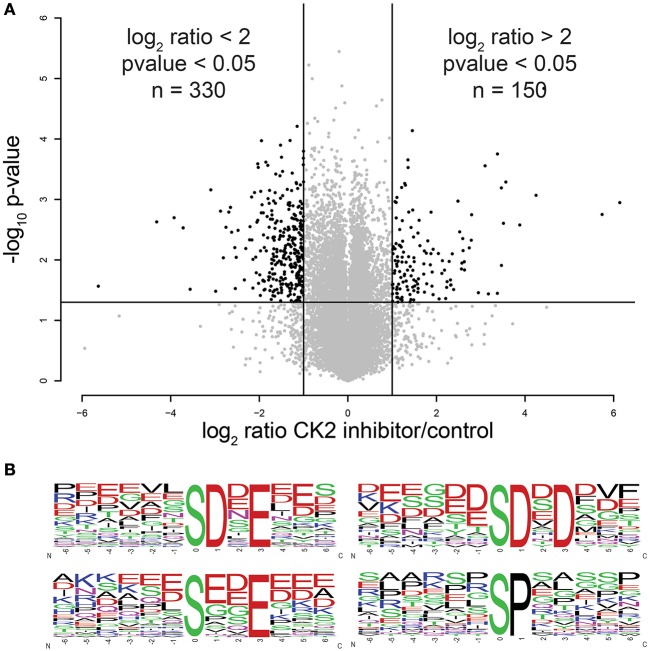
Statistical and motif analysis of candidate substrates. **(A)** Volcano plot of log_2_ phosphopeptide ratios vs. the negative log_10_ of the *p*-value of the replicate fold change. **(B)** Significantly enriched motifs from phosphorylation sites decreased by 2-fold or more upon CK2 inhibition.

Using this approach, we were able to identify 30,345 phosphopeptides corresponding to 23,995 unique phosphorylation sites on 5,060 proteins. Of these, 23,466 phosphopeptides corresponding to 18,603 unique phosphorylation sites on 4,306 proteins were quantified. The majority of phosphopeptides, 88%, were quantified in at least two of three biological replicates (Supplementary Table 1). To assess the reproducibility of quantification of phosphorylation site changes upon addition of CX-4945, we performed statistical analysis using Student's *T*-test (Figure 2A). In total 4,659 phosphopeptides were quantified with a *p* < 0.05. Of these statistically significant sites, 330 phosphopeptides corresponding to 287 phosphorylation sites on 202 proteins decreased by 2-fold or more (log_2_ ratio < −1), while 150 phosphopeptides corresponding to 144 phosphorylation sites on 116 proteins increased by 2-fold or more (log_2_ ratio >1) in abundance upon inhibition of CK2.

Next, we performed linear motif analysis of amino acids surrounding the phosphorylation site for sites that decreased in phosphorylation upon CK2 inhibition. In this analysis, we found an enrichment for an acidophilic motif with a glutamic or aspartic acid residue in the +1 and/or +3 positions downstream as well as a preference of acidic amino acids upstream of the phosphorylated residue (Figure 2B), which is consistent with the previously reported linear motif preference of CK2 (Marin et al., 1986; Meggio et al., 1994). We also detected a proline-directed motif in 14% of the significantly changed phosphopeptides (Figure 2B). It was previously reported that a proline-directed motif is incompatible with direct phosphorylation of CK2, but that proline-directed phosphorylation sites can prime for CK2 phosphorylation (Marin et al., 1992; St-Denis et al., 2015). Because most of the proline-directed phosphorylation sites that decreased upon CK2 inhibition were single phosphorylation sites, they are not likely to be part of a priming mechanism. Thus, we believe that these sites are either due to an off-target effect of CX-4945 or are an indirect result of CK2 inhibition and excluded these sites from consideration as candidate CK2 phosphorylation sites.

Among the sites identified as sensitive to CX-4945 treatment, several have been previously confirmed as *bona fide* CK2 substrates when compared to the PhosphositePlus database (Hornbeck et al., 2015). These candidate CK2 substrates included topoisomerase IIα (serine 1377), the translation initiation factor EIF2β (serine 2), the heat shock protein HSP90A serine 263), the elongation factor EF1D (serine 162), the histone deacetylase HDAC1 (serine 421 and serine 423), as well as the inhibitor of protein phosphatase 1 PPP1R2 (serine 121 and serine 122).

### Network analysis of candidate CK2 substrates

**Figure 3:**
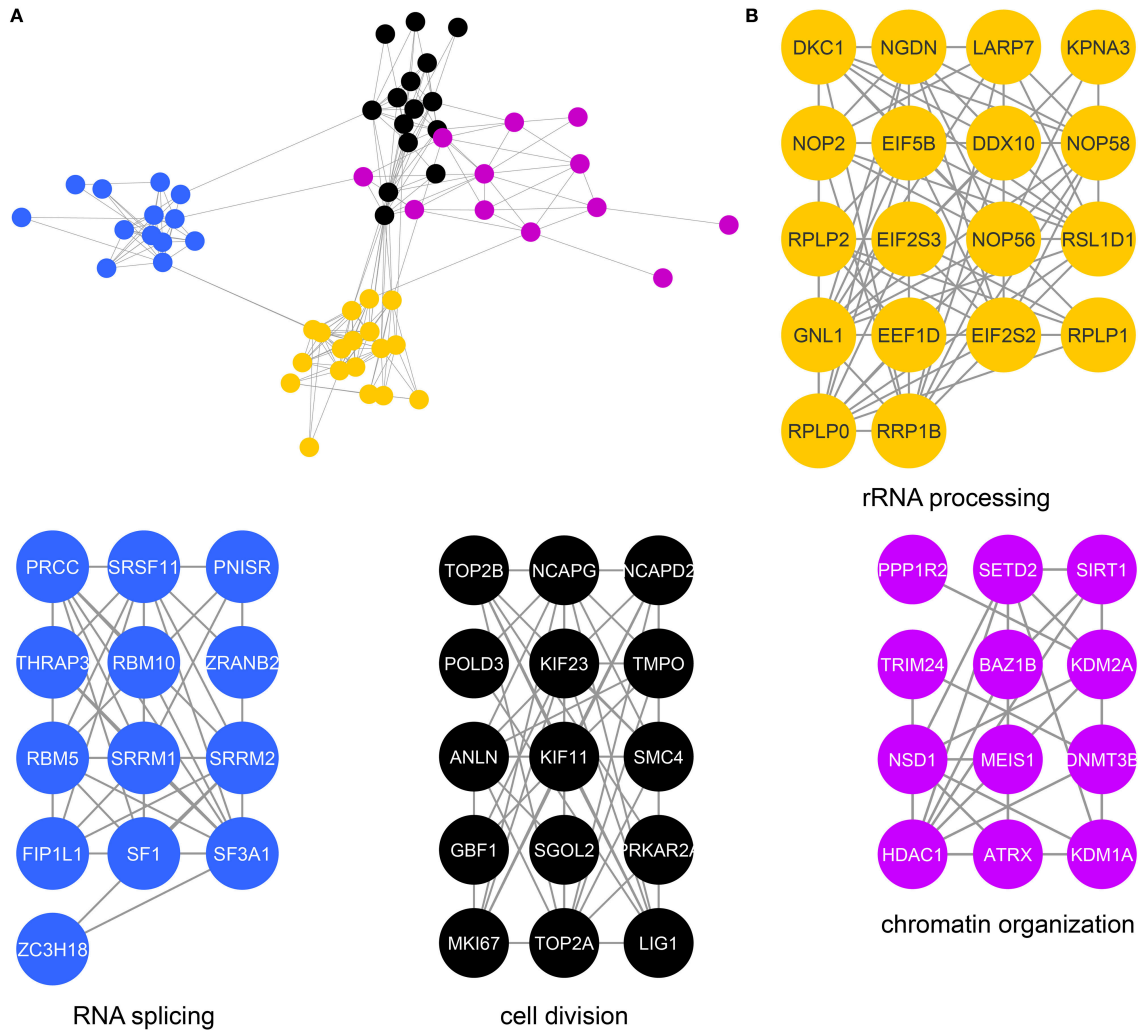
Protein interaction network of candidate mitotic CK2 substrates. **(A)** Network depicting statistically significant enriched clusters of interacting proteins from sites identified as potential CK2 substrates using STRING and visualized in Cytoscape. **(B)** Individual enriched clusters from **(A)** and their enriched Gene Ontology biological process.

We next determined specific biological processes regulated by CK2 in mitosis via network analysis of proteins that contain significantly decreased phosphorylation site with acidophilic motif. We gathered protein-protein interaction data from the STRING database (Search Tool for the Retrieval of Interacting Genes/Proteins) and analyzed these interactions in Cytoscape (Shannon et al., 2003; Saito et al., 2012; Szklarczyk et al., 2015). We identified highly connected clusters of proteins within the candidate substrates, which were enriched for specific biological processes (Figure 3A). The cluster in gold showed enrichment for rRNA processing and ribosomal biogenesis and contained proteins mainly localized to the ribonucleoprotein complexes (Figure 3B). In *Saccharomyces cerevisiae*, CK2 is a component of the pre-ribosome, where together with Tor1 it was implicated in pre-rRNA processing switching (Kos-Braun et al., 2017). This cluster also contains members of the EIF2 complex, including member EIF2β (EIF2S2) and EIF2S3. CK2 has been previously shown to be phosphorylated EIF2β on serine 2, a site also identified in our study, to activate the protein and stimulate mRNA production and cell growth (Llorens et al., 2006). We also identify another translation initiation factor, EIF5B, as a potential substrate of CK2. In *Saccharomyces cerevisiae* CK2 has been shown to phosphorylate EIF5, although the functions of this phosphorylation are currently unknown (Maiti et al., 2003). The blue cluster showed enrichment for RNA splicing and members of the spliceosomal complex. It has been previously shown that CK2 phosphorylation of proteins involved in catalyzing the splicing reaction increases their association with the spliceosome as well as the activity of the spliceosome itself (Kim et al., 2014). The cluster in black represents proteins that contribute to chromosome condensation that localize to chromosomes. Among those proteins are members of the condensin I complex, including NCAPG, NCAPD2, as well as SMC4, a protein shared between the condensin I and II complex. CK2 phosphorylation of condensin I complex members is known to inhibit its activity (Takemoto et al., 2006). In addition, we identify phosphorylation sites on both topoisomerase IIα, a known substrate of CK2 and topoisomerase IIβ, a known interactor of CK2β (Escargueil et al., 2000; Park et al., 2001). Interestingly the role of this specific phosphorylation site on topoisomerase IIα remains unknown. The fourth and final cluster in purple includes proteins involved with chromatin organization. Included in this group is the histone deacetylase HDAC1. CK2 phosphorylation of HDAC1 and HDAC2 is required for the dissociation of both proteins from each other and formation of HDAC1 and HDCA2 homodimers (Tsai and Seto, 2002; Khan et al., 2013). In our study we identify both sites on HDAC1 that have been implicated in this process, serine 421 and serine 423, as well as an additional phosphorylation site, serine 393, which has a currently unknown function. We also identify a phosphorylation site on the methyltransferase DNMT3B, S136. CK2 has been shown to phosphorylate DNM3TA and reduce its methyltransferase activity, however, the effect of CK2 phosphorylation of DNMT3B remains to be determined (Deplus et al., 2014). Also contained in this group is PPP1R2, a negative regulator of protein phosphatase PP1 and known substrate of CK2 as mentioned previously through database mining (Korrodi-Gregório et al., 2013). However, the precise role for these phosphorylation sites remains unknown.

### Analysis of CK2 interactome in mitotic HEK-293T cells

**Figure 4:**
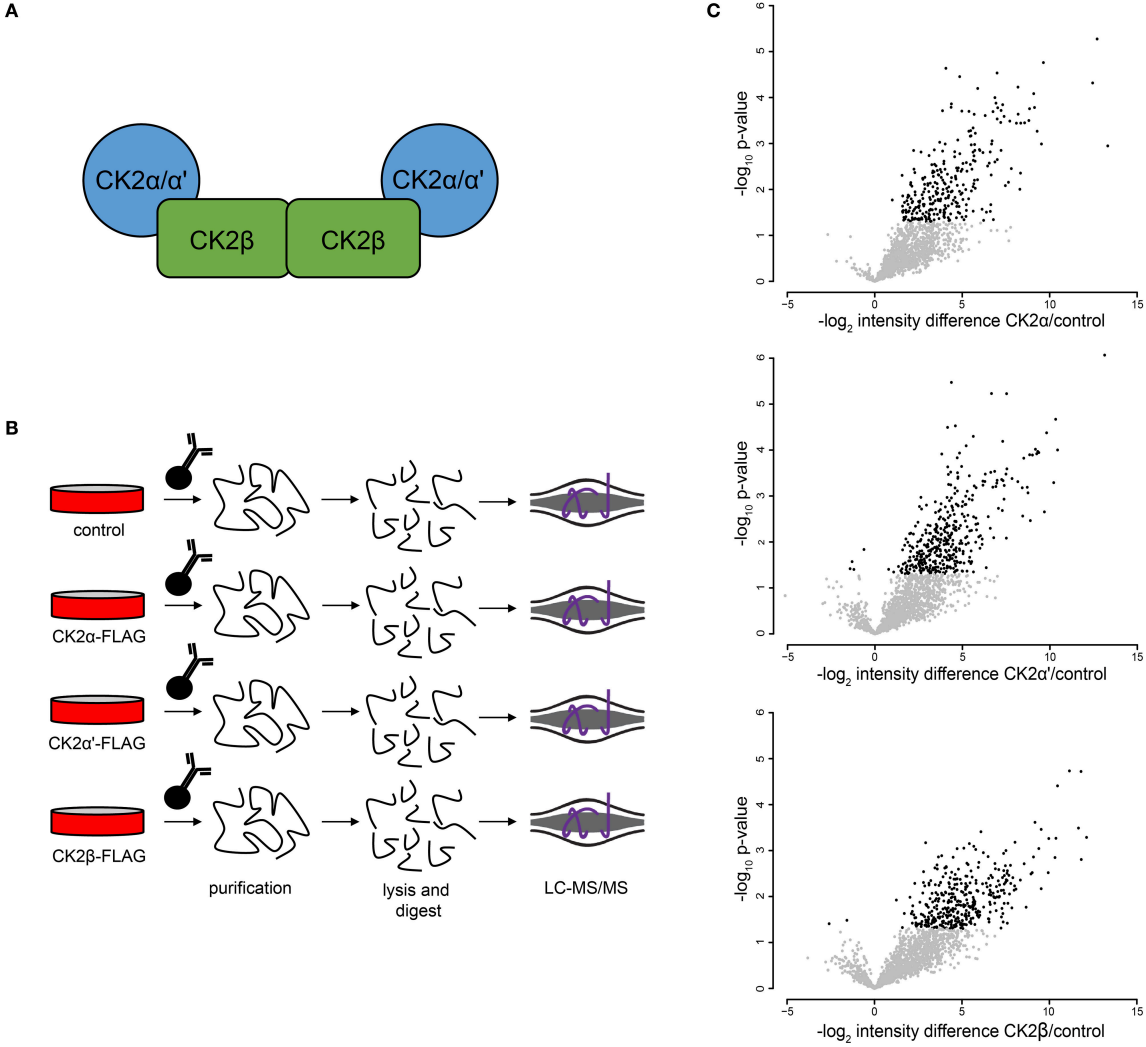
Mitotic CK2 AP-MS analysis. **(A)** Scheme depiction of tetrameric CK2 complex found in cells. **(B)** Scheme for identification of mitotic CK2 interactors. Cells were transfected with different FLAG-tagged CK2 subunits or control and mitotically arrested. Cells were collected, lysed, and FLAG-tagged proteins were purified, TCA precipitated, and analyzed by LC-MS/MS. **(C)** Volcano plots of the log_2_ difference in intensity between sample and control against the negative log_10_ of the *p*-value of the replicate fold change for each of the three purifications performed.

**Figure 5:**
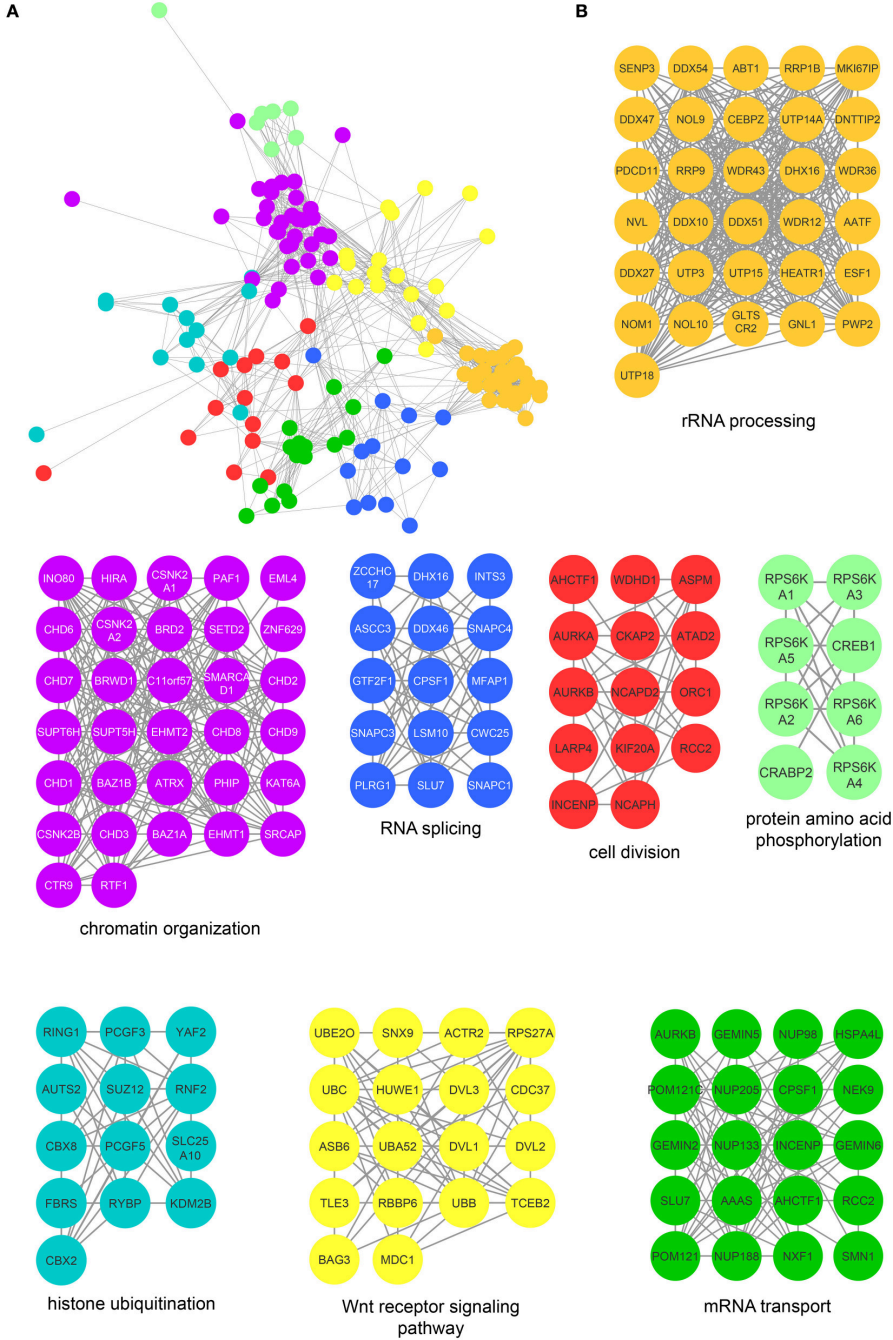
Protein interaction network of mitotic CK2 interactors. **(A)** Network depicting statistically significant enriched clusters of interacting proteins from CK2-interacting proteins identified by two or more peptides whose abundance increased 3-fold or more over control. **(B)** Individual enriched clusters from **(A)** and their enriched Gene Ontology biological process.

To further investigate CK2 regulated biological processes in mitosis, we identified proteins that interact with CK2 complex subunits in mitotic human cells. CK2 forms heterotetramers composed of two catalytic subunits (α or α', either as homo- or heterodimer) and two regulatory β subunits (Figure 4A). We generated FLAG-tagged CK2α, CK2α', and CK2β constructs, transiently transfected them into HEK-293T cells, arrested cells in mitosis with Taxol and performed affinity purification-mass spectrometry (AP-MS) analyses to determine interactors for each subunit (Figure 4B). All analyses were performed in quadruplicate. In these analyses, we identified 315 significant interactions compared to control for all subunits combined that were enriched 3-fold or more above control pull-downs (Figure 4C). Of those interactions the majority (184/315, 58.5%) interacted with only CK2β, with CK2α' having fewer (44/315, 14.1%) and CK2α having the fewest subunit-specific protein interactions (5/315, 0.2%) (Supplementary Table 2). The other interacting proteins (82/315, 26.1%) were identified in AP-MS analyses of two or more subunits. We then investigated CK2 interactors using the STRING database and Cytoscape for their connectivity and identified eight distinct nodes (Figure 5A).

The most highly enriched cluster (gold) included proteins involved in rRNA processing (Figure 5B). Several interactors in this group were also identified as substrates (Figure 3B), including the RNA helicase DDX10, the guanine nucleotide binding protein GNL1 and RRP1B, a ribosomal RNA processing protein which has been linked as a susceptibility marker in breast cancer (Lee et al., 2014). The second most significant set of interactors (purple) harbored proteins involved in chromatin organization, again overlapping with proteins identified as substrates (Figure 3B). For instance, BAZ1B and SETD2 are candidate substrates as well as interactors. In addition, we identified a large number of DNA helicases [chromodomain-helicase-DNA-binding-protein (CHD) 1, 2, 3, 6, 7, 8, and 9]. These proteins play key roles in chromatin remodeling, a process that CK2 has been implicated in regulating in *Saccharomyces cerevisiae* (Barz et al., 2003). These proteins also interact with another protein in this group, SUPT6H, which itself has been shown to interact with IWS1, a putative CK2 substrate identified in our screen, and implicated in mRNA processing (Yoh et al., 2007). While no phosphorylation sites were identified for SUPT6H or the CHD proteins identified as interactors, it is possible that these proteins could target CK2 to IWS1 to promote phosphorylation. A smaller cluster of interacting proteins (blue) belongs to proteins involved in RNA splicing, which was also overlapping with enriched biological processes identified in the substrate analysis. Here, we identified members of the small nuclear RNA-activating protein (SNAP) complex, including SNAPC1, SNAPC3 and SNAPC4. It was previously demonstrated that CK2 phosphorylates SNAPC4 and this phosphorylation leads to loss in SNAPC4 DNA binding and loss of U6 transcriptional activity (Gu et al., 2007). A number of general splicing factors were also identified, such as SLU7, DHX16, and MFAP1, which again agrees with a general role for CK2 in mRNA splicing (Lehnert et al., 2008). Another cluster of tightly-associated interactors (red) included proteins involved in cell division, for instance members of the condensin I complex including (NCAPD2 and NCAPH). We also identify several proteins with known roles in spindle dynamics, including Aurora A kinase (AURKA), the kinesin KIF20A, and the microtubule binder ASPM (Neef et al., 2003; Floyd et al., 2008; Jiang et al., 2017). ASPM is identified as both an interactor and a candidate substrate of CK2, with phosphorylation of serine 35 decreasing upon loss of CK2 activity. We also identified INCENP and Aurora B, key members of the chromosomal passenger complex (CPC), which associates with the kinesin KIF20A (Hümmer and Mayer, 2009). Previous work has shown that CK2 localizes to the mitotic spindle through interaction with the peptidyl proline isomerase Pin1 (St-Denis et al., 2011). A smaller cluster of interacting proteins (light green) included six members of the RSK (α1-6) (90 kDa ribosomal ribosomal S6 kinase) family of protein kinases which is important for cell growth and differentiation. CK2 has been shown to phosphorylate RPS6KA2 and facilitate its nuclear export (Panasyuk et al., 2006). Consistent with this report, we find that RPS6KA2 as well as the other five members of the RSK family only interact with the β subunit. Another subset of interactors (cyan) includes members of the Polycomb repressive complexes (PRC1 and PRC2), including chromobox protein homologs 2 and 8 of PRC1 and SUZ12 of PRC2. CK2 has been shown to phosphorylate members of PRC1 resulting in unconventional transcriptional activation by PRC1 (Gao et al., 2014). However, the interaction and potential regulatory function of CK2 and PRC2 are new, and a role for CK2 in activating or repressing PRC2 has not been described to date. We also identified a cluster of enriched in proteins (yellow) involved in Wnt pathway signaling, including Wnt signal relay disheveled (DVL). CK2 has been shown to interact with and phosphorylate DVL3 resulting in the generation of a docking motif for CK1δ/ε, which further phosphorylates and activates DVL3 (Bernatik et al., 2011). This cluster also contained the E3-ubiquitin ligase Huwe1, a candidate CK2 substrate (serine 2887), which was previously not implicated in CK2 signaling. Finally, we discovered a group of interactors that function in RNA transport (dark green). This group included nuclear pore proteins (NUP98, NUP133, NUP185 and NUP205), mRNA export factors, such as NXF1 and other mRNA processing proteins SLU7, CPSF1, and SMN1.

### CK2–PP6 opposition on shared substrates

**Figure 6:**
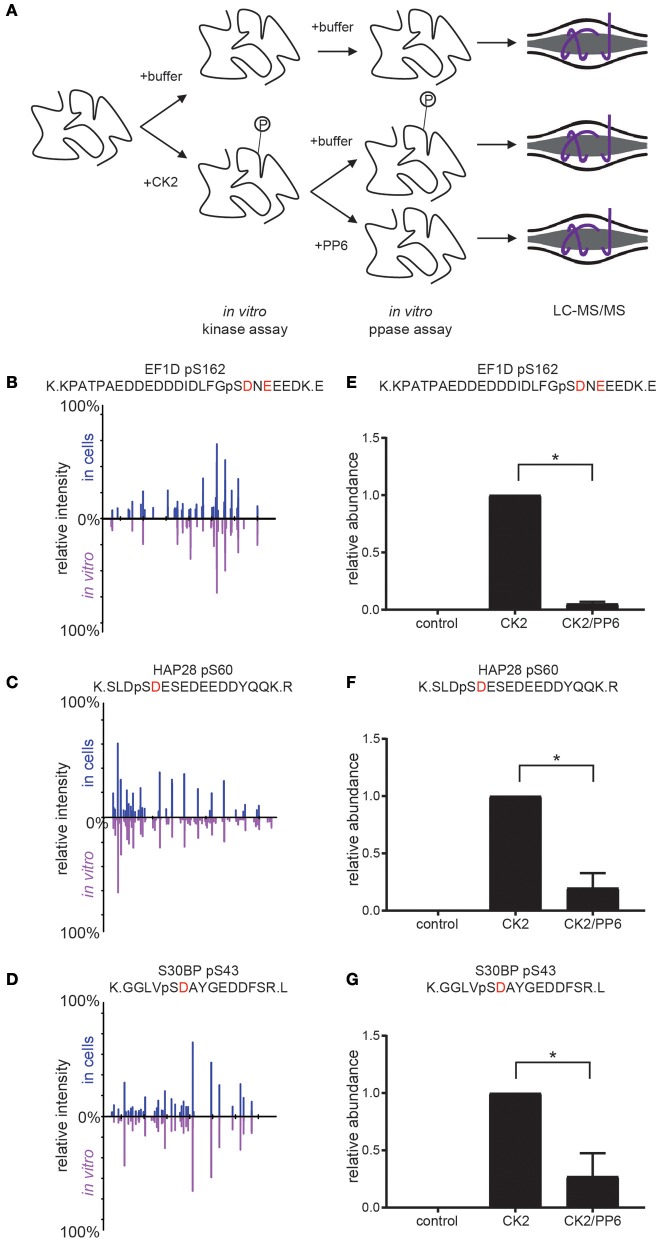
Coupled *in vitro* kinase and phosphatase workflow and results. **(A)** Workflow for coupled *in vitro* kinase and phosphatase assays. Purified substrates were incubated with or without purified CK2 in *in vitro* kinase assay buffer at 28°C for 2 h and dialyzed overnight against *in vitro* kinase assay buffer without ATP. Substrates were then incubated with or without purified PP6 in *in vitro* phosphatase buffer at 28°C for 2 h, reduced, separated by SDS-PAGE, Coomassie stained, excised, digested with protease, and analyzed by LC-MS/MS. **(B–D)** Aligned reciprocal tandem mass spectra results for candidate CK2/PP6c substrates from *in vivo* (top, blue) and *in vitro* (bottom, purple) analyses. **(E–G)** Quantification of phosphorylation sites abundance in control, CK2 and CK2 and PP6 treated conditions using Students *T*-test, ^*^p < 0.05, *n* = 2 independent experiments for EF1D, 3 for others.

In previous work, we have shown that CK2 phosphorylates serine 973/serine 975 on the condensin I subunit NCAPG resulting in its inactivation and that PP6 opposes CK2 on these sites, resulting in condensin I dephosphorylation and activation at the beginning of mitosis (Rusin et al., 2015). Furthermore, we found that PP6 preferentially dephosphorylates serine and threonine residues proximal to acidic residues (similar to the preference for CK2 (SxxE) (Rusin et al., 2015). To extend these findings and to validate specific candidate CK2 substrates, we performed coupled *in vitro* kinase—phosphatase assays using purified PP6 and CK2 holoenzyme and candidate substrates (Figure 6A).

For these analyses, we selected proteins with phosphorylation sites that were surrounded by an acidophilic linear kinase motif downstream of the phosphorylatable amino acid and significantly decreased upon CK2 inhibition as well as significantly increased upon PP6c depletion (EF1D, HAP28, S30BP) (Rusin et al., 2015). We generated his-tagged CK2α and untagged CK2β, then coexpressed them and purified CK2 complexes from *Spodoptera frugiperda* (Sf9) cells. Substrates were expressed in bacteria as his-tagged constructs. Purified CK2 and substrates were combined in the presence or absence of ATP and incubated at 28°C for 2 h in *in vitro* kinase assay buffer. Reactions were dialyzed overnight at 4°C against *in vitro* kinase assay buffer without ATP. Purified PP6c was then added or not and reactions were incubated at 28°C for 2 h. Reactions were quenched, resolved by SDS-PAGE, gels were Coomassie blue stained and analyzed by LC-MS/MS (Figure 6A). We found that CK2 was able to phosphorylate the candidate CK2 phosphorylation sites detected in our screen *in vitro* and that the MS2 spectra for these sites match in both the *in vivo* screen and *in vitro* kinase assays (Figures 6B–D). Upon addition of PP6, we detected a significant decrease in phosphorylation of these sites (Figures 6E–G). Consistent with our previous findings that PP6 was capable of counteracting CK2 phosphorylation of serine 973/serine 975 on the condensin I subunit NCAPG, PP6 was capable of dephosphorylating other serine and threonine sites proximal to acidic residues in other CK2 substrates, suggesting a more common regulatory mechanism.

### Effect of CK2α overexpression on chromosome condensation

**Figure 7:**
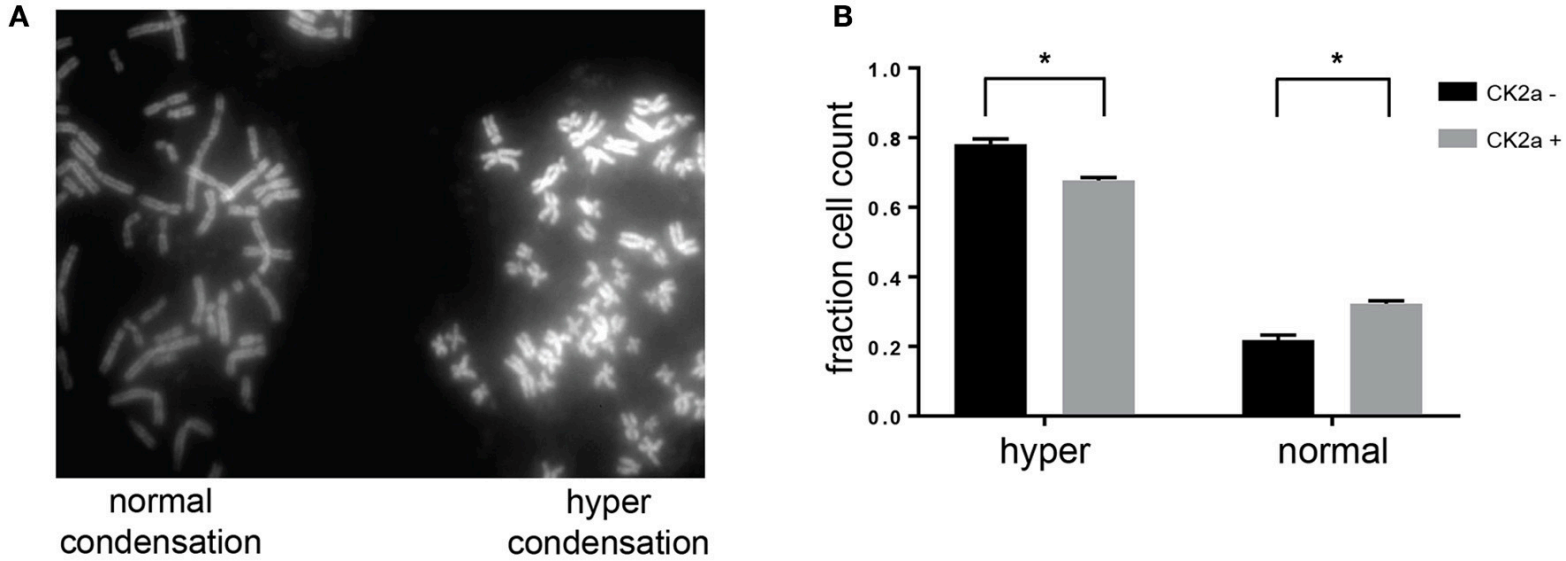
Effects of CK2 on condensin I phosphorylation and activity. **(A)** Representative image from chromosome spreads depicting normally (left) and hyper (right) condensed chromosomes. **(B)** Quantification of differences in chromosome condensation between cells with and without overexpression of CK2α, ^*^p < 0.05, *n* = 3 independent experiments.

Previous work from our laboratory and others has shown a role for CK2 in the negative regulation of chromosome condensation through phosphorylation of members of the condensin I complex (Takemoto et al., 2006; Rusin et al., 2015). CK2-dependent phosphorylation of condensin I inhibits its ability to condense chromosomes using a *Xenopus laevis in vitro* system (Takemoto et al., 2006). Given the identification of several members of the condensin I complex as candidate substrates and interactors of CK2, we determined the effect of CK2 overexpression on chromosome condensation in HeLa cells. To do so, we stably introduced inducible, myc-tagged CK2α into HeLa cells. Upon addition of doxycycline, expression of CK2α was induced within 12 h. However, the expression level of exogenous CK2 was low compared to endogenous levels (Supplementary Figure 2). To determine if a slight increase in CK2α would affect chromosome condensation, we treated cells with nocodazole for 4 h to arrest them in mitosis and performed chromosome spreads. Cells arrested for prolonged periods of time in mitosis undergo hypercondensation of chromosomes due to the continued action of condensin I. Upon overexpression of CK2α, we detected a small but statistically significant decrease in the percentage of cells with hypercondensed chromosomes, mirroring effects seen with either loss of condensin I members or though inactivation of PP6, which opposes CK2 phosphorylation resulting in the activation of condensin I (Figure 7; Hirota et al., 2004; Rusin et al., 2015).

## Discussion

Accurate segregation of genetic material and cellular organelles in mitosis is crucial for the generation of two identical daughter cells. Many of the processes that contribute and control chromosome and organelle segregation are orchestrated via phosphorylation by protein kinases and dephosphorylation by protein phosphatases. Here, we add to the existing knowledge of mitotic kinases and their substrates by interrogating mitotic cells for candidate substrates of the kinase CK2. Using the selective CK2 inhibitor CX-4945 and quantitative mass spectrometry-based phosphoprotemics, we identify 330 phosphorylation sites on 202 proteins as candidate CK2 substrates (Supplementary Table 1). The majority of these sites contained an acidophilic linear kinase motif consistent with the canonical CK2 motif (pS/pT-D/E-X-D/E) (Figure 2). Among others, we were able to identify several previously established CK2 substrates in our screen, including EF1D, eIF2β, HAP28, Topoisomerase IIα and HDAC1 (Shen et al., 1996; Gyenis et al., 2011) confirming the validity of our approach. Candidate CK2 substrates were enriched in biological processes, such as rRNA processing and RNA splicing, cell division and chromatin organization. Consistently, the CK2 interactome was also enriched in these four biological processes among others (Figure 5). Interestingly, we identified candidate CK2 phosphorylation sites on 20 of the proteins identified as specific interactors of CK2 subunits (Supplementary Tables 1, 2). We also confirmed CK2 phosphorylation of specific substrates by *in vitro* kinase assay (Figure 6) and show a role for CK2 in chromosome condensation in cells.

CK2 has been implicated in the regulation of a plethora of biological processes ranging from transcription and translation to cell survival and cell cycle progression, however, its role in mitosis is still emerging (Nñnez de Villavicencio-Diaz et al., 2017). Our analysis has revealed many new candidate CK2 substrates that need to be explored in future investigations to elaborate the role of CK2 in mitosis. While our analysis was conducted under mitotic arrest condition, it is possible that CK2 phosphorylates some of these candidate substrates also in other cell cycle phases. In contrast to other mitotic kinases, including Aurora kinase A and B, Polo-like kinase 1, and Cyclin-dependent kinase 1, whose activities are tightly controlled and limited to mitosis, CK2's activity is thought to be constitutively active (Pinna, 2002; van de Weerdt and Medema, 2006; Salaun et al., 2008; Turowec et al., 2010). Thus, candidate mitotic substrate expressed in other cell cycle phases could be phosphorylated by CK2 outside of mitosis.

Understanding mitotic progression requires not only knowledge of mitotic kinase substrates and their temporal and spatially resolved phosphorylation patterns, but also the counteraction and reversal of these phosphorylation sites by protein phosphatases. While research efforts over the last decade have revealed many kinase substrates, counteracting phosphatases and the shared substrates that link kinases and phosphatases are often elusive. The majority of dephosphorylation in interphase as well as mitosis is carried out by the family of phosphoprotein phosphatases (PPP) (Shi, 2009). We have previously reported that PP6, but not PP2A, opposes CK2 on specific phosphorylation sites (Rusin et al., 2015). We further extend these observations here, showing that opposition of CK2 by PP6 might be a more general mechanism. This is of significant interest, as no other member of the PPP family has been described so far to dephosphorylate acidophilic sites (Wurzenberger and Gerlich, 2011). Furthermore, a counteraction of the *Saccharomyces cerevisiae* homologs of PP6c and casein kinase, Sit4 and Hrr25, respectively, has been described for the regulation of the elongator complex (Mehlgarten et al., 2009). Connecting kinases and phosphatases on their shared substrates is essential for completing our understanding of cellular signaling. Follow-up studies of these candidate substrates are necessary to further clarify CK2–PP6 dependent substrate interactions and establish the biological significance of these findings.

In summary, the work performed here expands upon the current knowledge of CK2 substrates and functions in mitosis and potentially other cell cycle phases, and provides a resource to begin exploring the largely unknown role of this prolific kinase in a crucial cell cycle phase.

## Author contributions

SR performed the CK2 inhibitor phosphoproteomics and interactome experiments as well as *in vitro* kinase and phosphatase assays and chromosome spreads. MA managed data files and the data pipeline for all proteomics experiments and generated software for iBAQ quantification. AK designed the study. SR and AK analyzed the data. SR generated the figures. SR and AK wrote the manuscript.

### Conflict of interest statement

The authors declare that the research was conducted in the absence of any commercial or financial relationships that could be construed as a potential conflict of interest.

## Supplementary material

Supplementary Figure 1 Experimental design of CK2 phosphoproteomic analysis.

Supplementary Figure 2 Inducible CK2α protein expression in HeLa FLP cells. Western blot using α-CK2α antibody antibody of HeLa FLP and HeLa FLP- CK2α expressing cells in the presence and absence of doxycycline.

Supplementary Table 1 Data of phosphoproteomics analysis of CX-4945 treated, mitotically-arrested HeLa cells.

Supplementary Table 2 Data of CK2 AP-MS analysis.
