# Supplementary figures and images for: Identification of Candidate Casein Kinase 2 Substrates in Mitosis by Quantitative Phosphoproteomics
Source: Front Cell Dev Biol. 2017 Nov 22;5:97. doi: 10.3389/fcell.2017.00097 (PMC5702644; doi:10.3389/fcell.2017.00097)

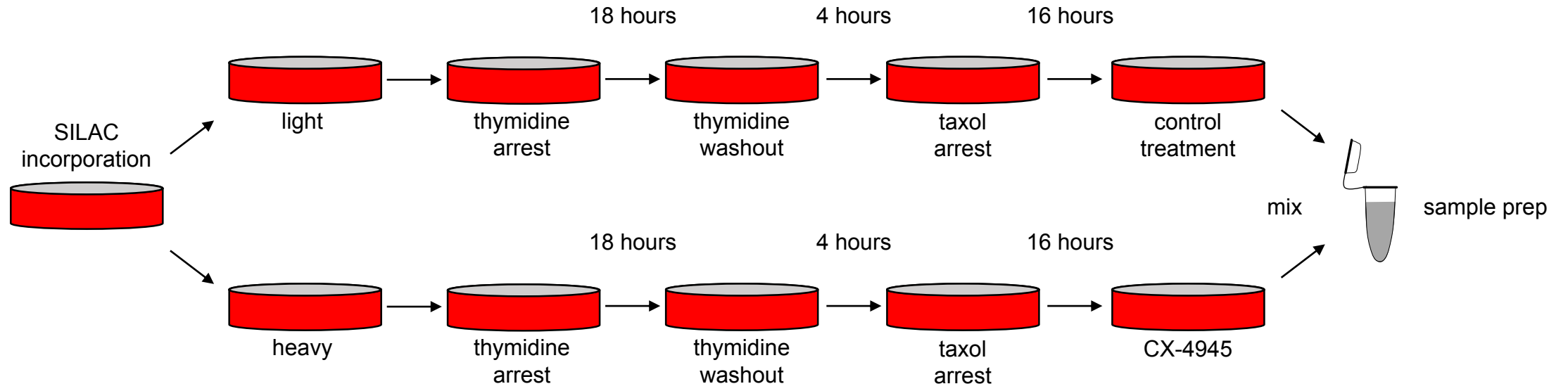

Supplementary Figure 1

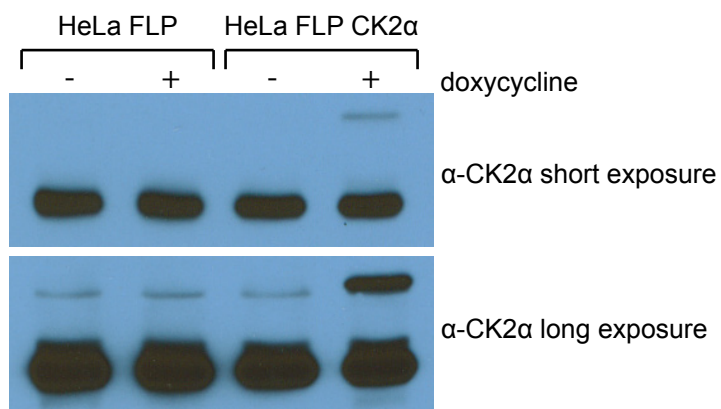

Supplementary Figure 2

Supplement: Supplementary Figure 1 — Experimental design of CK2 phosphoproteomic analysis. [file Image1.PDF]
